# Unexpected diagnosis of sarcoidosis on bone marrow trephine biopsy

**DOI:** 10.1002/jha2.127

**Published:** 2020-12-11

**Authors:** Stewart Hunt, Camille Savoia, Shannon Emmett

**Affiliations:** ^1^ Department of Haematology Gold Coast University Hospital Queensland Australia

A 53‐year‐old female was referred to hematology by her GP for self‐detected splenomegaly and fatigue. She had no other constitutional symptoms and no significant past medical history. The only pertinent finding on physical examination was splenomegaly, four‐finger breadths below the left costal margin. Full blood count and film were unremarkable. Positron emission tomography/computed tomography showed mildly avid bilateral hilar and mediastinal lymph nodes (some with internal calcification), multiple mildly avid portocaval, para‐aortic, aortocaval, and left iliac nodes. Spleen was 16.8 cm with SUVmax 14.7 and multiple avid osseous lesions were seen.

Bone marrow aspirate and trephine were performed as part of the work‐up for a lymphoproliferative disorder. The aspirate was normocellular with normal trilineage hematopoiesis. The trephine was hypercellular with marked distortion of architecture and effacement of normal hematopoietic tissue (Figure 1, low power). There werenumerous well‐formed epithelioid granulomas, which contained Langerhans‐type giant cells without necrosis and lacked surrounding inflammatory cells (see Figure 2, low power). There was prominent reticulin fibrosis and collagen formation surrounding the granulomas (see Figure 3, reticulin). Immunohistochemistry with CD68 demonstrated histiocytes/macrophages within the granulomas (see Figure 4, CD68). Various microbiology stains on the trephine and blood serology were negative for infectious causes. Serum angiotensin converting enzyme was elevated at 262 μg/L. A bronchoscopy was performed for completeness and was unremarkable.

**FIGURE 1:**
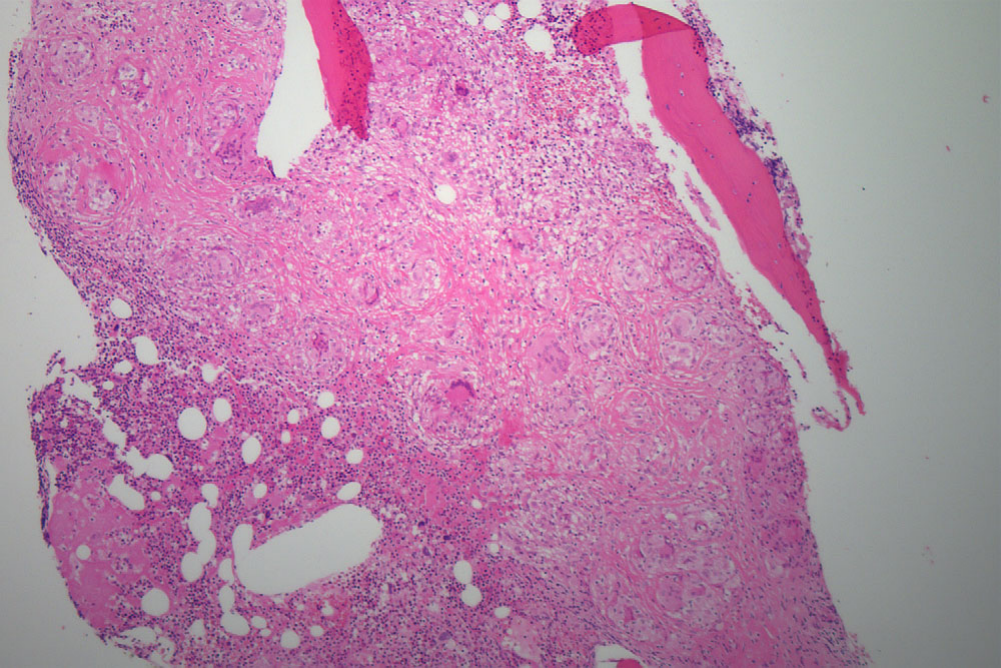
Bone marrow trephine HE stain under low power

**FIGURE 2:**
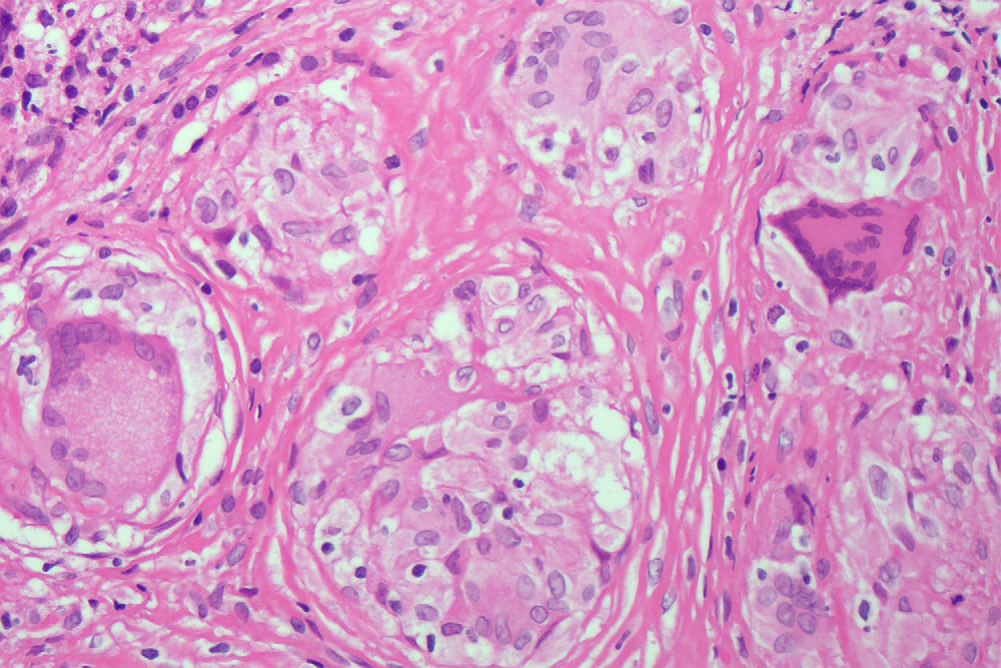
Bone marrow trephine HE stain under high power

**FIGURE 3:**
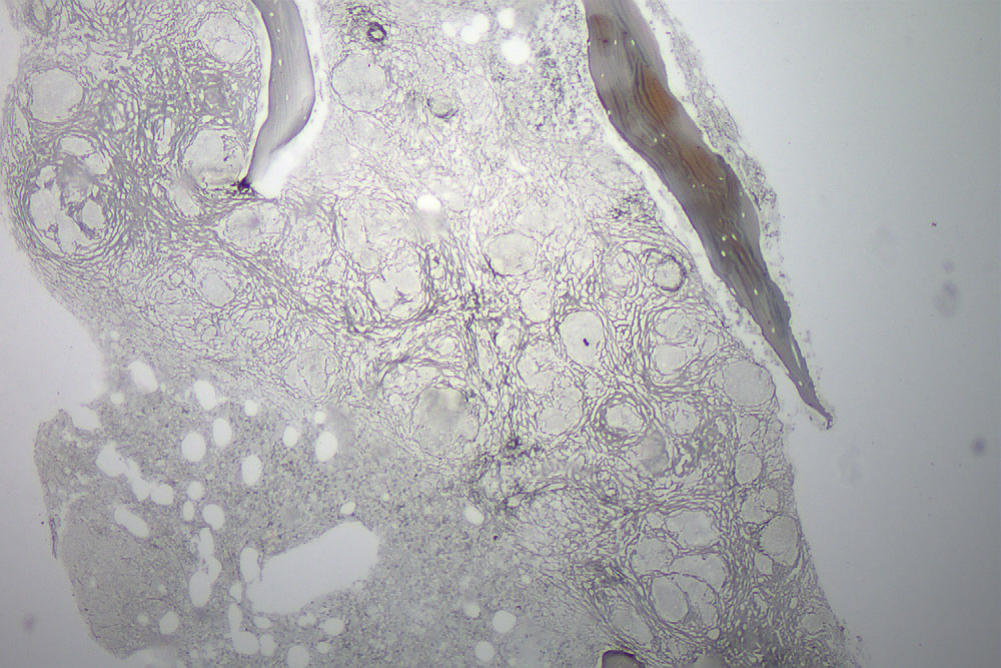
Bone marrow trephine reticulin stain under low power

**FIGURE 4:**
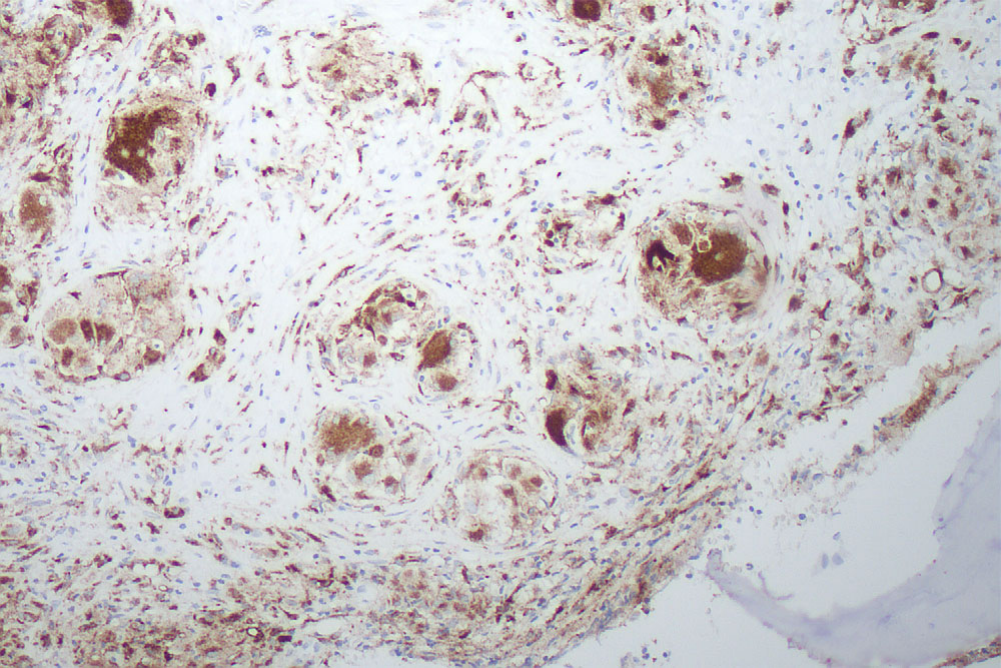
Bone marrow trephine stained with CD68 immunohistochemistry

Sarcoidosis is a rare multisystem disorder that is characterized by the presence of noncaseating granulomas. Pulmonary disease and symptoms are the most common presentation. While bone marrow involvement can be common in sarcoidosis, this case demonstrates diffuse involvement and was diagnostic for this patient.

## AUTHOR CONTRIBUTIONS

All three authors wrote and reviewed the submission.

